# Management of Hereditary Transthyretin Amyloidosis (ATTRv) Patients and Asymptomatic Carriers in Spain: The EMPATIa Study

**DOI:** 10.3390/jcm13247587

**Published:** 2024-12-13

**Authors:** Inés Losada López, Juan Gonzalez-Moreno, Juan Buades Reinés, Teresa Sevilla, Fernando Martinez Valle, Lucía Galán Dávila, Francisco Muñoz Beamud, José Eulalio Bárcena Llona, Manuel Romero Acebal, Patricia Tarilonte, Francesca Setaro

**Affiliations:** 1 Internal Medicine Department, Hospital Universitario Son Llàtzer, Palma de Mallorca, 07198 Palma, Spain; 2 Balearic Research Group in Genetic Cardiopathies, Sudden Death and TTR Amyloidosis, Instituto de Investigación Sanitaria de las Islas Baleares (IdISBa), 07198 Palma, Spain; 3 Neurology Department, Hospital Universitari i Politècnic La Fe & IIS La Fe Universitat de València, 46010 Valencia, Spain; 4 Centro de Investigación Biomédica en Red de Enfermedades Raras (CIBERER), 28034 Madrid, Spain; 5 Internal Medicine Department, Hospital Universitario Vall d’Hebron, 08035 Barcelona, Spain; 6 Neurology Department, Hospital Clínico San Carlos, IdISSC, 28040 Madrid, Spain; 7 Internal Medicine Department, Hospital Universitario Juan Ramón Jiménez, 21005 Huelva, Spain; 8 Neurology Department, Hospital Universitario Cruces, 48903 Barakaldo, Spain; 9 Neurology Department, Hospital Universitario Virgen de la Victoria, 29010 Málaga, Spain; 10 Pfizer S.L.U., 28108 Madrid, Spain

**Keywords:** transthyretin, amyloidosis, ATTRv mutation, management

## Abstract

**Background:** Hereditary transthyretin amyloidosis (ATTRv) is an autosomal-dominant systemic disease, where amyloid fibrils accumulate especially in the peripheral and autonomic nervous systems and in the heart. The aim of the present work was to outline the follow-up and type of management received by asymptomatic carriers (ACs) and *Coutinho* stage 1 ATTRv patients in Spain. **Methods:** A cross-sectional, non-interventional study was conducted throughout seven experienced hospitals in Spain. A total of 86 ACs without neurological symptoms and 19 *Coutinho* stage 1 ATTRv patients diagnosed 12 months before their enrollment were included. Clinical and demographic data, red flags, and neurological and cardiological evaluations were gathered. In addition, site variables were collected from four centers to describe the clinical management of ATTRv. **Results:** ATTRv clinical management varied depending on the center setting but was primarily overseen by neurology and internal medicine, which were responsible for the holistic follow-up of ACs and patients. Routinely, neurologists, neurophysiologists, cardiologists, and internal medicine conducted the follow-up. Specialties involved in initial AC assessment were neurophysiologists and cardiologists in 100% of cases, neurologists (75%), internists and geneticists (50%), and ophthalmologists (25%). A review of the medical tests performed proved an exhaustive management of the study population. Stable patients were followed up every 6 months, while those under evolution were monitored every 3–6 months. The frequency of monitoring of ACs was annual, and carriers classified with doubtful disease onset were visited every 3–6 months. **Conclusions:** The EMPATIa study provides valuable insights into the management of ATTRv in a real-world clinical setting in highly experienced hospitals in Spain. It demonstrates that multidisciplinary practice and enhanced disease awareness may lead to a reduction in diagnostic delay.

## 1. Introduction

Hereditary transthyretin amyloidosis (ATTRv) is an autosomal-dominant systemic disease with variable penetrance, marked by the deposit of transthyretin (TTR) in several locations of the body. So far, more than 150 distinct genetic mutations have been observed in the *TTR* gene [1,2]. The overall prevalence of ATTRv is less than 1 case per 100,000 inhabitants, for what is considered a rare disease, but there are some endemic areas where the prevalence is higher (Portugal, Japan, Sweden, and Brazil) [3]. Specifically, two high-prevalence (endemic) areas exist in Spain, Mallorca (Balearic Islands) and Valverde del Camino (Huelva) [4].

TTR is a mainly liver-synthesized protein that is also produced in the choroid plexus and retinal pigment epithelium. TTR, in tetramer form, is responsible for transporting the thyroid hormone thyroxine (T_4_) and retinol (vitamin A) through the plasma and cerebrospinal fluid. In fact, ATTR is a product of TTR tetramer dissociation. TTR monomers experience conformational changes, producing non-native oligomers and amyloid fibrils [5]. These amyloid fibrils accumulate in different tissues, particularly targeting the peripheral and autonomic nervous systems and the cardiac muscle [2], leading to neuropathy and cardiomyopathy [6], but also in the eyes or kidneys [7], ultimately leading to a systemic dysfunction [8].

The EMPATIa study aimed to describe the characteristics and management of *Coutinho* stage 1 ATTRv patients with polyneuropathy (PN) and asymptomatic carriers (ACs) of a *TTR* gene mutation from endemic and non-endemic areas in Spain. Recently, the clinical profile description of these populations has been published, confirming the multisystemic clinical profile of the disease across Spain [9]. Here, the mean diagnostic delay was 1.8 years, shorter than the 3–4 years reported in earlier studies [10,11].

Diagnosis remains a challenge due to the lack of distinctive symptoms and the incomplete penetrance of the disease, with late diagnosis directly impacting the early initiation of therapies and prognosis [7]. A systemic approach and multidisciplinary evaluation may enable an early diagnosis by means of the identification of initial clinical manifestations, also known as “red flags” [7,12,13]. In this regard, the present study outlines the follow-up and management approaches provided to *TTR* gene mutation ACs and *Coutinho* stage 1 ATTRv-PN patients within the EMPATIa study.

## 2. Materials and Methods

### 2.1. Study Population

Eligibility criteria for the EMPATIa study have previously been described [9]. Briefly, this study included ACs without PN symptoms related to ATTRv and *Coutinho* stage 1 ATTRv-PN patients diagnosed in the 12 months prior to the recruitment. Exclusion criteria included individuals with diabetes mellitus, *Coutinho* stage 2/3 ATTRv, monoclonal gammopathy of undetermined significance, pregnant or breastfeeding woman, and those who did not provide informed consent.

### 2.2. Study Design

EMPATIa is a multicenter, cross-sectional, non-interventional, descriptive epidemiological study carried out at seven experienced hospitals from endemic (Huelva and Mallorca) and non-endemic areas (Madrid, Barcelona, Vizcaya, Málaga, and Valencia) in Spain. The conception and validation of the study protocol were performed before the last publication of guidelines [14], meaning that the established criterion for discriminating between ACs and patients was based on the presence of PN, mostly diagnosed on the basis of large fiber involvement by electromyogram. However, by clinical decision, 3 subjects presenting autonomic dysfunction without sensory-motor PN were also classified as patients [9]. This trial was conducted in accordance with the Declaration of Helsinki. The study protocol was approved by the Clinical Research Ethics Committee of Vall d’Hebron Hospital (Barcelona). All patients provided written informed consent.

### 2.3. Clinical Management

Although seven centers participated in the EMPATIa study, only four centers included data on ACs and patient management during the study period. In order to describe the clinical management and organizational setting of centers, the following variables were collected for each participating site: total number of ACs monitored in the center and maximum time and frequency of follow-up, specialists involved in baseline and follow-up visits, tests performed, and scales employed at diagnosis and/or follow-up for both ACs and ATTRv patients.

The following assessments were compiled: (i) sensory-motor and autonomic neurological impairment using electromyography (EMG); (ii) study of autonomic nervous system by scales (COMPASS 31); (iii) cardiovascular autonomic dysfunction by the use of the Tilt test and the RR interval; (iv) sudomotor function and electrochemical skin conductance (ESC) on the SUDOSCAN device (Impetomedical, Issy-les-Moulineaux, France); (v) the ATTRv-related neurological status determined by the neuropathy impairment score (NIS); (vi) the quality of life (QoL) by using the scale of the Norfolk Quality of Life Questionnaire for Diabetic Neuropathy (Norfolk QoL-DN); (vii) comorbidities by means of Charlson comorbidity index (CCI); (viii) cardiological study by the use of electrocardiogram (ECG) and echocardiogram (ECHO); and (iv) laboratory data, including serum biomarkers such as the N-terminal prohormone of brain natriuretic peptide (NT-proBNP), troponin T and I, albumin and transthyretin levels, and evidence of microalbuminuria and proteinuria.

### 2.4. ATTRv Amyloidosis Red Flags

All data collected were obtained from electronic medical records and recorded in an electronic case report form (eCRF). As previously described, ATTRv red flags were collected and categorized as follows: autonomic and peripheral sensory-motor neuropathies; central nervous system (CNS) manifestations; ocular, kidney, gastrointestinal, and cardiac alterations; and carpal tunnel syndrome [9,12]. Autonomic neuropathy is defined as orthostatic hypotension, recurrent urinary tract infections due to urinary retention, sexual dysfunction, and sweating disturbances. All AC red flags recorded in the eCRD from the seven centers included in this study were analyzed to identify potential differences based on the frequency of follow-up.

### 2.5. Statistical Analysis

A descriptive statistical analysis was conducted for all variables, including measures of central tendency and dispersion for continuous variables (median and interquartile range [IQR] or mean and standard deviation [SD]) and absolute and relative frequencies for categorical variables. Student’s *t*-test and the Mann–Whitney U test were used to compare quantitative variables, and Chi-square (χ2) or Fisher tests for qualitative variables. Tests were two-tailed with a significance level of 5%.

## 3. Results

### 3.1. Clinical Management of Hereditary Transthyretin Amyloidosis

At the time of this study, 138 ACs were under follow-up in the study centers. The mean number of ACs followed up in the four included centers was 34.5 (range 15–84), 46.1% were male; the age of ACs ranged between 19 and 81 years old, and the mean age at *TTR* gene mutation identification was 42.9 years old (range 41–45).

**Figure 1:**
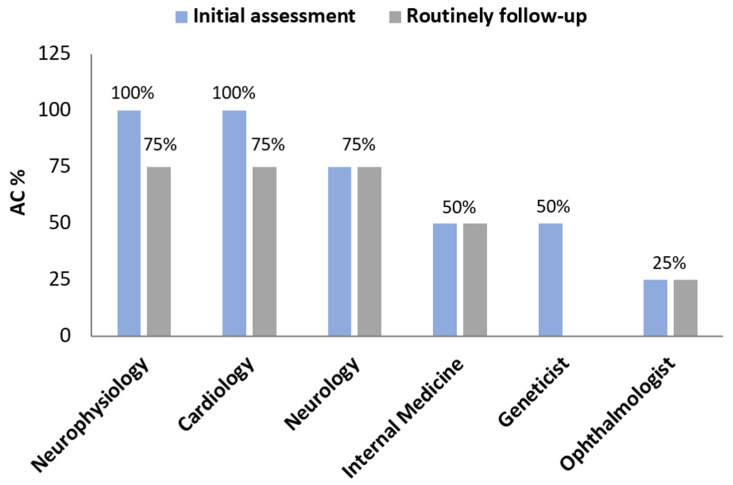
Specialists involved in initial assessment and routine follow-up of asymptomatic carriers (ACs).

Regarding mutations, 100% of the sites followed up carriers with V50M; 75% of sites, carriers with S97Y mutation; and 50% of the centers, mutations V142I and E109K. Other mutations reported were T60A, D38E, and T68I. Initial AC evaluation was carried out by neurophysiologists and cardiologists (all centers), neurologists (75% of centers), internist physicians and geneticists (half of the centers), and ophthalmologists (25% of centers), while the routine follow-up monitoring was primarily driven by specialists in neurophysiology, neurology, and cardiology (75%) and internist physicians (50%) (Figure 1).

**Figure 2:**
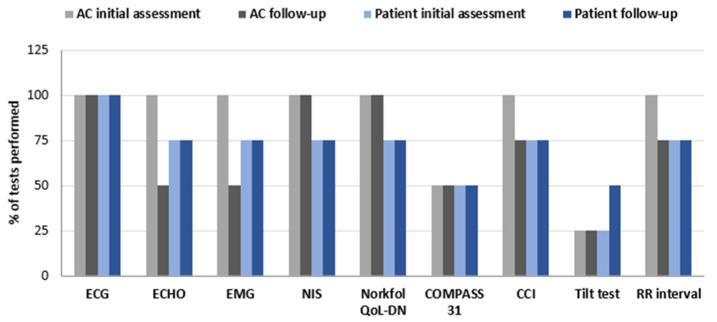
Frequency of use of clinical tools for the first assessments and in the follow-up of patients and asymptomatic carriers. AC: asymptomatic carrier; ECG: electrocardiogram; ECHO: echocardiogram; EMG: electromyography; NIS: neuropathy impairment score; CCI: Charlson comorbidity index; Norfolk QoL-DN: Norfolk Quality of Life Questionnaire for Diabetic Neuropathy.

Interestingly, a careful review of the medical tests and scales used for the initial evaluation and follow-up demonstrated an exhaustive and integrative management of the study population (Figure 2). The initial AC visit was the most complete, and all centers included ECG, ECHO, EMG, NIS, Norfolk-QoL-DN, CCI, and RR interval; the COMPASSS-31 scale was used in 50% of centers. In the successive medical examinations, ECG, NIS, and Norfolk-QoL-DN were performed by all centers, and RR interval was used in 75% of centers, while ECHO, EMG, and COMPASS-31 were routinely performed in 50% of centers among other available tests. The frequency of monitoring was annual, and in the case of a carrier classified with doubtful disease onset, the frequency of visits was every 3–6 months.

With respect to patients’ initial assessment, all centers performed ECG; 75% included ECHO, EMG, NIS, Norfolk QoL-DN, CCI, and RR interval; and 50% included COMPASS-31. At follow-up, the same tests and scales were used in the same percentages in all centers as in the first patient visit, except for the Tilt test (Figure 2). The frequency of follow-up was every 6 months for stable patients and every 3–6 months for patients under evolution (unstable).

### 3.2. ACs Characteristics According to Follow-Up Frequency

In order to determine whether there were different/specific characteristics among included ACs (n = 86) who require more or less follow-up, a cut-off of 12 months was applied. As mentioned above, annual monitoring was the predominant type of follow-up inside this population (n = 56 [71.8%]; follow-up frequency every 3 [n = 1], 6 [n = 20], 9 months [n = 1]). We found non-statistical differences in the AC age subgroups, with the subgroup monitored with a lower frequency being younger (43.6 ± 15.1 years) than ACs more frequently monitored (49.3 ± 11.8 years; 0 = 0.074). Within the group of subjects followed up yearly, 85.7% (n = 48) were V50M, 10.7% (n = 6) were S97Y, and only 1.8% (n = 1) were V142I or T60A. The group monitored with a frequency < 1 annual visit was significantly enriched in V142I mutation (1.8 % vs 27.3%; *p* = 0.006); 50% (n = 11) of subjects were V50M, 13.6% (n = 3) were S97Y, and 9.1% (n = 2) were D38E. No significant differences were found in anamnesis or red flags between carrier subgroups (Table 1).

## 4. Discussion

The EMPATIa study focused on the characterization of *TTR* gene mutation ACs and *Coutinho* stage 1 ATTRv patients in order to evaluate the early stages of the disease and to gain further insight into the management that these patients receive in a real-world clinical practice scenario where healthcare professionals have extensive experience with this disease. In this regard, an analysis of the multidisciplinary assessment and follow-up performed in both ACs and ATTR-PN patients was carried out.

The time to reach a diagnosis in ATTRv varies substantially between endemic (1 year) and non-endemic areas (3–4 years), and disease awareness plays a crucial role in reducing diagnostic delays [11]. In fact, different studies showed a high number of visits to hospital services of ATTRv patients during the 3 years prior to diagnosis as well as an important deterioration in their QoL during the same period [15,16]. The EMPATIa study showed that the overall diagnostic delay reported across experienced centers in Spain was 1.8 years, confirming again that a high level of knowledge on ATTRv allows the identification of patients in early stages [9]. A coordinated and systematic follow-up of ACs, involving multiple disciplines, is crucial for the early detection of symptoms, facilitating timely treatment and delaying disease progression [7,17,18], a recommendation that is also met in the clinical practice of the Spanish centers analyzed. Moreover, AC education is essential to guarantee their collaboration in the early identification of the first disease signs and symptoms that appear [7].

Since symptomatic ATTRv is associated with an increased morbidity and mortality, and induces a substantial impairment in patients’ QoL, it is vital to perform a proper follow-up adapted to the course and severity of the disease and therefore the response to the treatment. Clinical guidelines recommend that patients be monitored every 6–12 months [19]. In the centers participating in this study, we have confirmed that ATTRv management was carried out according to international guidelines [19]. Indeed, a comparative analysis with international standards revealed that the Spanish centers analyzed applied an intensified follow-up since stable patients were commonly monitored by a specialist every 6 months, while patients under evolution were monitored every 3–6 months, independently of whether care was provided in endemic or non-endemic areas [17,19]. In addition, ACs monitored more frequently than yearly were slightly older than ACs monitored every 12 months, probably because their predicted age of onset of symptomatic disease was approaching. We could also speculate that the shorter time to diagnosis described in these centers [9] might be attributed to more frequent follow-up. The EMPATIa results highlighted the importance of adapting the follow-up schedule case by case, according to mutation and familiar clinical history.

Follow-up testing and assessments for ACs should be tailored to the expected phenotypic presentation associated with each specific mutation. In terms of recommended clinical tools for the management of identified ACs [7], the EMPATIa study showed alignment with recommended techniques to identify potentially earlier signs of the disease, incorporating follow-up questionnaires for sensorimotor and autonomic symptoms, neurological examination, neurophysiological evaluations, autonomic functional testing, and relevant cardiac investigations.

Disease progression has effects at the systemic level and in several domains, such as functional capacity, QoL, levels of laboratory biomarkers, etc., the interpretation of which requires multidisciplinary expertise [20]. Therefore, healthcare professionals need a trustworthy regular assessment to identify early signals of disease appearance and to assess disease evolution in order to make suitable therapeutic decisions on an individual basis [18]. In EMPATIa-participating hospitals, ATTRv was overseen and referred by a single professional (neurologist or internist). However, the study results highlighted that initial assessment and follow-up visits were conducted by different specialties, ensuring holistic care of both populations, with follow-up varying slightly between centers due to the casuistry of each hospital. A timely and precise diagnosis provides patients with prolonged life expectancy, prevents hospitalizations, and saves costs for the national health system [21].

## 5. Conclusions

The EMPATIa study described accurately a representative population of ACs and *Coutinho* stage 1 ATTRv-PN patients with early stages of neurological impairment diagnosed in the last 12 months in seven Spanish hospitals, offering an overview of ATTRv management in a real-world clinical practice setting. The results confirmed that extensive disease awareness and integrated multidisciplinary management of ACs and patients based on a systematic approach and tailored to each clinical case is crucial for the early detection of the disease and therefore for improving patient care. We hope that this knowledge may contribute to the improvement of AC/ATTRv patient handling and guide clinicians in making suitable therapeutic decisions.

## Figures and Tables

**Table 1:** Red flags detected in asymptomatic carriers according to follow-up frequency.

	>1 Visit per Year		≤1 Visit per Year		*p*-Value
	N	%	N	%	
**CNS alterations**	3	13.6	3	5.4	0.342
**Ophthalmic alterations**	0	0.0	3	5.4	0.555
**Cardiac alterations**	3	13.6	5	8.9	0.680
**Carpal tunnel syndrome**	4	18.2	6	10.7	0.455
**Gastrointestinal alterations**	1	4.5	9	16.1	0.267
**Autonomic neuropathy**	5	22.7	6	10.7	0.276

CNS: central nervous system.

## Data Availability

The datasets used during the current study are not openly available due to reasons of sensitivity and are available from the corresponding author on reasonable request.

## Abbreviations

AC	asymptomatic carrier
ATTR	transthyretin amyloidosis
ATTRv	hereditary transthyretin amyloidosis
EMPATIa	Carrier Management Study in Transthyretin hereditary Amyloidosis (from the Spanish: “Estudio sobre el Manejo de los Portadores en Amiloidosis hereditaria por Transtiretina”)
ESC	electrochemical skin conductance
ECG	electrocardiogram
ECHO	echocardiogram
NORFOLK QoL-DN	Norfolk quality of life questionnaire for diabetic neuropathy
NIS	neuropathy impairment score
NT-proBNP	N-terminal prohormone of brain natriuretic peptide
QoL	quality of life
PN	polyneuropathy
TTR	transthyretin

## References

[1] Ueda M. (2022). Transthyretin: Its function and amyloid formation. Neurochem. Int..

[2] Nativi-Nicolau J.N., Karam C., Khella S., Maurer M.S. (2022). Screening for ATTR amyloidosis in the clinic: Overlapping disorders, misdiagnosis, and multiorgan awareness. Heart Fail. Rev..

[3] Condoluci A., Theaudin M., Schwotzer R., Pazhenkottil A.P., Arosio P., Averaimo M., Bacher U., Bode P., Cavalli A., Dirnhofer S. (2021). Management of transthyretin amyloidosis. Swiss Med. Wkly..

[4] Garcia-Pavia P., Dominguez F., Gonzalez-Lopez E. (2021). Transthyretin amyloid cardiomyopathy. Med. Clin..

[5] Yee A.W., Aldeghi M., Blakeley M.P., Ostermann A., Mas P.J., Moulin M., de Sanctis D., Bowler M.W., Mueller-Dieckmann C., Mitchell E.P. (2019). A molecular mechanism for transthyretin amyloidogenesis. Nat. Commun..

[6] Dispenzieri A., Coelho T., Conceicao I., Waddington-Cruz M., Wixner J., Kristen A.V., Rapezzi C., Plante-Bordeneuve V., Gonzalez-Moreno J., Maurer M.S. (2022). Clinical and genetic profile of patients enrolled in the Transthyretin Amyloidosis Outcomes Survey (THAOS): 14-year update. Orphanet. J. Rare Dis..

[7] Conceição I., Damy T., Romero M., Galán L., Attarian S., Luigetti M., Sadeh M., Sarafov S., Tournev I., Ueda M. (2019). Early diagnosis of ATTR amyloidosis through targeted follow-up of identified carriers of TTR gene mutations. Amyloid.

[8] Ibrahim R.B., Liu Y.T., Yeh S.Y., Tsai J.W. (2019). Contributions of Animal Models to the Mechanisms and Therapies of Transthyretin Amyloidosis. Front. Physiol..

[9] Galan Davila L., Martinez Valle F., Buades Reines J., Gonzalez-Moreno J., Losada Lopez I., Sevilla T., Munoz Beamud F., Barcena Llona J.E., Romero Acebal M., Setaro F. (2024). A description of variant transthyretin amyloidosis (ATTRv) stage 1 patients and asymptomatic carriers in Spain: The EMPATIa study. Orphanet. J. Rare Dis..

[10] Adams D., Ando Y., Beirão J.M., Coelho T., Gertz M.A., Gillmore J.D., Hawkins P.N., Lousada I., Suhr O.B., Merlini G. (2021). Expert consensus recommendations to improve diagnosis of ATTR amyloidosis with polyneuropathy. J. Neurol..

[11] Adams D., Koike H., Slama M., Coelho T. (2019). Hereditary transthyretin amyloidosis: A model of medical progress for a fatal disease. Nat. Rev. Neurol..

[12] Conceição I., González-Duarte A., Obici L., Schmidt H.H.J., Simoneau D., Ong M.-L., Amass L. (2016). “Red-flag” symptom clusters in transthyretin familial amyloid polyneuropathy. J. Peripher. Nerv. Syst..

[13] Losada I., Gonzalez-Moreno J., Rodriguez A., Uson M., Ripoll-Vera T., Ferrer-Nadal A., Rigo E., Andreu H., Figuerola A., Montala J.C. (2020). Multidisciplinary approach in the management of hATTR. Eur. J. Clin. Investig..

[14] Maurer M.S., Bokhari S., Damy T., Dorbala S., Drachman B.M., Fontana M., Grogan M., Kristen A.V., Lousada I., Nativi-Nicolau J. (2019). Expert Consensus Recommendations for the Suspicion and Diagnosis of Transthyretin Cardiac Amyloidosis. Circ. Heart Fail..

[15] Lane T., Fontana M., Martinez-Naharro A., Quarta C.C., Whelan C.J., Petrie A., Rowczenio D.M., Gilbertson J.A., Hutt D.F., Rezk T. (2019). Natural History, Quality of Life, and Outcome in Cardiac Transthyretin Amyloidosis. Circulation.

[16] López-Sainz Á., Hernandez-Hernandez A., Gonzalez-Lopez E., Domínguez F., Restrepo-Cordoba M.A., Cobo-Marcos M., Gómez-Bueno M., Hernandez-Perez F.J., Oteo J.F., Mirelis J.G. (2021). Clinical profile and outcome of cardiac amyloidosis in a Spanish referral center. Rev. Esp. Cardiol. (Engl. Ed.).

[17] Adams D., Suhr O.B., Hund E., Obici L., Tournev I., Campistol J.M., Slama M.S., Hazenberg B.P., Coelho T., European Network for T.-F. (2016). First European consensus for diagnosis, management, and treatment of transthyretin familial amyloid polyneuropathy. Curr. Opin. Neurol..

[18] Conceição I., Coelho T., Rapezzi C., Parman Y., Obici L., Galán L., Rousseau A. (2019). Assessment of patients with hereditary transthyretin amyloidosis—Understanding the impact of management and disease progression. Amyloid.

[19] Ando Y., Adams D., Benson M.D., Berk J.L., Plante-Bordeneuve V., Coelho T., Conceicao I., Ericzon B.G., Obici L., Rapezzi C. (2022). Guidelines and new directions in the therapy and monitoring of ATTRv amyloidosis. Amyloid.

[20] Garcia-Pavia P., Bengel F., Brito D., Damy T., Duca F., Dorbala S., Nativi-Nicolau J., Obici L., Rapezzi C., Sekijima Y. (2021). Expert consensus on the monitoring of transthyretin amyloid cardiomyopathy. Eur. J. Heart Fail..

[21] Formiga F., Garcia-Pavia P., Martin Sanchez F.J., Navarro-Ruiz A., Rubio-Terres C., Peral C., Tarilonte P., Lopez-Ibanez de Aldecoa A., Rubio-Rodriguez D. (2021). Health and economic impact of the correct diagnosis of transthyretin cardiac amyloidosis in Spain. Expert Rev. Pharmacoecon Outcomes Res..

